# Dermatologic and Ophthalmologic Treatment of Erythema Multiforme Major: A Case Report

**DOI:** 10.7759/cureus.20854

**Published:** 2021-12-31

**Authors:** Barbara Senger, Shayan A Memar, Alex Ahmann, Jeremy J Houser, Lauren Doughty-McDonald

**Affiliations:** 1 Internal Medicine, A.T. Still University, Kirksville College of Osteopathic Medicine, Kirksville, USA; 2 Anatomy, A.T. Still University, Kirksville College of Osteopathic Medicine, Kirksville, USA; 3 Dermatology, Dermatology Clinic of Iowa, Cedar Rapids, USA

**Keywords:** ocular complications, nsaids, mycoplasma pneumonia, hsv-1, erythema multiforme major

## Abstract

Erythema multiforme major (EMM) is a rare type IV cytotoxic reaction targeting keratinocytes of the mucosal surfaces and the dermis. Dusky, targetoid lesions with central clearing are classically present, which may become blistered and rupture. The disease is usually self-limited and managed with supportive care and treatment of the underlying condition. The most common triggering factors are adverse reactions to medications, herpes simplex virus (HSV), and *Mycoplasma pneumoniae*. Rapid recognition of EMM is essential to avoid long-term complications. This case presents a 39-year-old male with a unique history of recent non-steroidal anti-inflammatory drug (NSAID) use, past infection with HSV-1, and an acute *Mycoplasma pneumoniae* infection. The patient developed painful lesions on the skin, oral mucosa, ocular surfaces, and urethra. The painful lesions caused complications with feeding and voiding. Initially, the triggering event was unclear. Supportive care was started. NSAIDs were discontinued and similarly-structured drugs were avoided. Treatments targeting *Mycoplasma pneumoniae* and HSV-1 were initiated while lab results were pending. Once the results returned, the treatment regimen of corticosteroids for inflammation, acyclovir for HSV-1, and azithromycin for *Mycoplasma pneumoniae* was continued. Vaseline was applied to open lesions. The patient was also treated with mouthwash consisting of aluminum (Al) hydroxide/magnesium (Mg) hydroxide/simethicone (400 mg/400 mg/40 mg). Topical 2% lidocaine gel with applicator was used to assist with urinary discomfort during voiding. Fentanyl was used for pain control. The patient successfully recovered and was discharged to follow-up with ophthalmology. Long-term sequelae including trichiasis, symblepharon, and punctal stenosis were noted during follow-up appointments.

## Introduction

Erythema multiforme (EM) is a type IV cytotoxic reaction mainly targeting keratinocytes, resulting in classic targetoid lesions [1]. EM was once thought to be a part of spectrum of disorders including Steven-Johnson syndrome (SJS) and toxic epidermal necrolysis (TEN). However, EM is now recognized as a distinct disease [2]. Histopathologically, EM differs from SJS/TEN. EM consists of T cell lymphocytic infiltrate, while SJS/TEN are characterized by necrotic tissue infiltrated by macrophages and dendritic cells [3]. EM has a vast variety of etiologies including herpes simplex virus (HSV), *Mycoplasma pneumoniae*, fungal infections, and medications (penicillin, sulfonamides, non-steroidal anti-inflammatory drugs {NSAIDs}, and barbiturates) [2,4-7].

EM has two variants, erythema multiforme major (EMM) and erythema multiforme minor (EMm). EMM and EMm are both characterized by cutaneous lesions affecting <10% of the body surface area. The lesions are targetoid with blistered or necrotic centers, surrounded by an erythematous ring. They are acute and usually resolve with the potential for scarring [8]. The differentiating factor between EMM and EMm is mucosal surface involvement; EMM affects ≥1 mucosal surface, while EMm has minimal to no mucosal involvement [7,9]. The oral mucosa (70%) is the most common mucosal site involved; however, other surfaces including the eyes, genitals, upper respiratory, and pharyngeal mucosa may also be affected [2,8]. According to Chang et al., ocular involvement in patients with EM is uncommon (22.7%) compared to SJS (81.3%) and TEN (66.7%) [10]. Since rare, serious ocular sequelae include symblepharon, conjunctival recession, corneal erosions, and/or trichiasis, thus it is critical for ophthalmology to be consulted upon an EM/SJS/TEN diagnosis [10].

This report details a 39-year-old male who was diagnosed with EMM. In addition to skin lesions, this case is unique due to the ocular, oral, and urethral mucosal surfaces being affected. He had reported recent NSAID use. He was positive for IgG antibodies (5.27 index) to HSV-1. He also had IgM and IgG (1.94 Units/L and 2.24 Units/L) antibodies to *Mycoplasma pneumoniae*. The patient responded well to antivirals, antibiotics, steroids, opioids, mouthwash, and ophthalmic treatment.

## Case presentation

A 39-year-old Caucasian male presented to the emergency department (ED) with lip and mouth sores and drainage from both eyes. Five days prior to lesion onset, he experienced a fever, fatigue, a sore throat, and malaise. Upon onset of the constitutional symptoms, he began taking ibuprofen once daily. The lesions started in his mouth as ulcers with hemorrhagic crusting. The lesions progressed to the extremities, abdomen, and back as numerous erythematous papules with dusky centers and interspersed vesicles. The patient reported decreased oral intake. He experienced similar symptoms twice in the past after taking NSAIDs, but the lesions were less severe and self-limiting. He denied a history of insect bites or stings, denied previous outbreak of HSV, and was unaware of *Mycoplasma pneumoniae* contact. He also denied any past medical problems and did not take any medications except ibuprofen. His family history was significant for a brother with uncontrollable pemphigus vulgaris.

**Figure 1:**
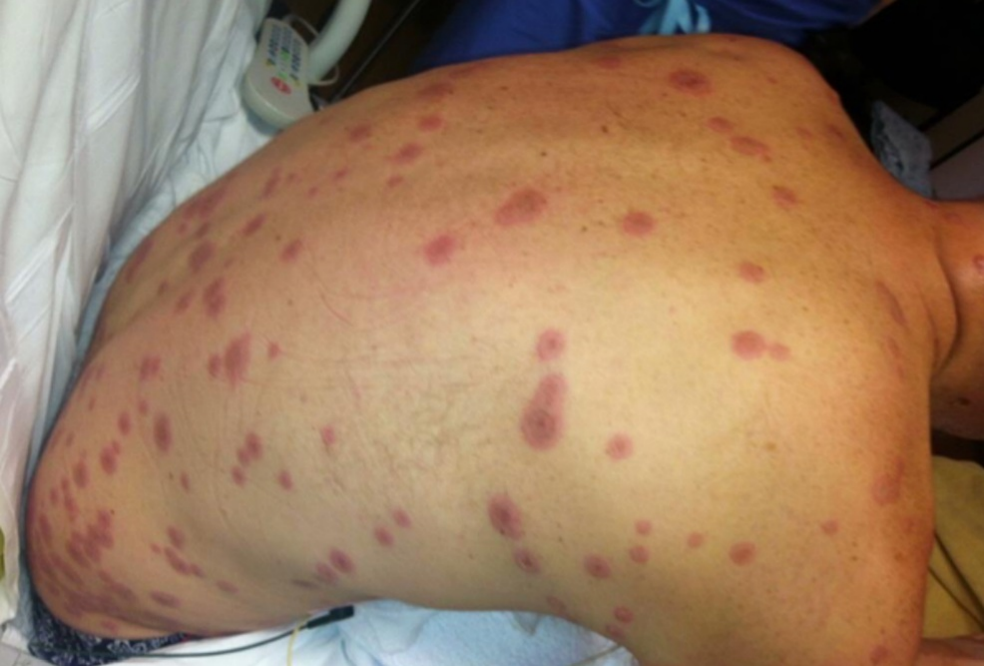
Numerous targetoid EMM lesions spread diffusely on the back. EMM: erythema multiforme major

**Figure 2:**
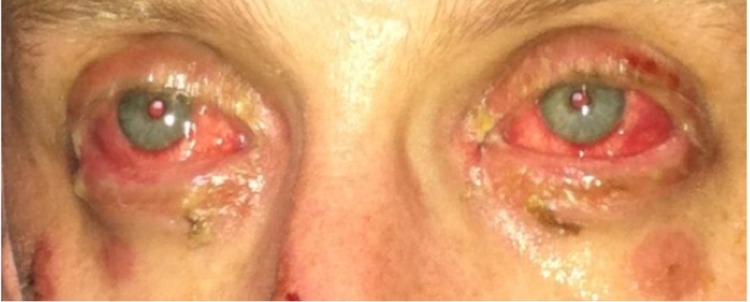
Bilateral conjunctivitis with injected sclera secondary to EMM. EMM: erythema multiforme major

**Figure 3:**
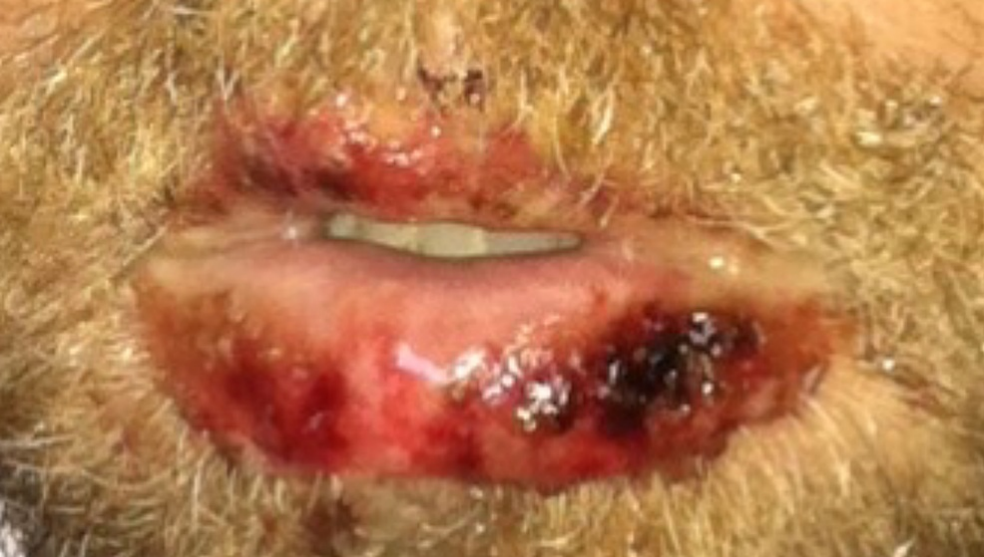
Sloughing of oral labia secondary to EMM. EMM: erythema multiforme major

**Figure 4:**
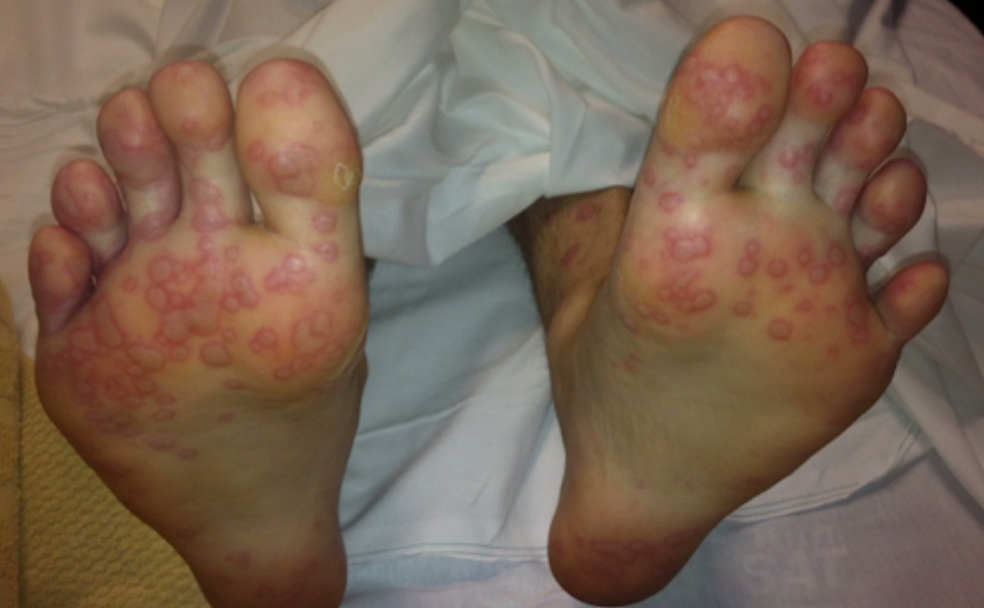
Cutaneous lesions on the plantar surfaces secondary to EMM. EMM: erythema multiforme major

During the initial examination at the ED, the patient was alert and in no acute distress. His vitals were as follows: a temperature of 36°C, a heart rate of 113 beats per minute (bpm), a respiratory rate of 16 breaths per minute, and a blood pressure of 118/76 mmHg. Upon physical examination, the skin was warm, dry, and pink with multiple 5 mm macules, some with areas of central clearing, spread diffusely across the upper extremities, abdomen, chest, and back (Figure 1). Bilateral conjunctivitis, clear drainage, chemosis, and injected sclera were present (Figure 2). Oral mucosa was dry with appreciable skin sloughing on the upper and lower lips with no crusting (Figure 3). Cardiovascular and pulmonary examinations were unremarkable. Dermatology and ophthalmology were consulted on this patient. After 24 hours, the lesions evolved to erythematous papules with dusky centers and some interspersed vesicles on his back, chest, abdomen, left palm, and bilateral dorsal eyelids. Erosions on the mucosal lip and punched-out erythematous lesions with cobblestoning of the hard palate were noted. There was significant staining in both eyes with fluorescein with no abrasions. The patient experienced dysuria, with no mucosal involvement of the urethra. After 48 hours, the lesions spread to the scalp, face, neck, bilateral lower extremities, palms, dorsal hands, dorsal feet, and soles (Figure 4). Erosions on the mucosal lip and the hard palate remained present. The patient was unable to tolerate oral intake due to extreme pain with swallowing. At this time, there were erosions and erythema in the urethral meatus. The eyelids were edematous with erythematous conjunctiva, but no corneal involvement. The patient denied any photophobia or vision changes. After 72 hours, a majority of the skin lesions stabilized and regressed over the remainder of the hospital stay. There was aggressive mattering and conjunctival injection with bilateral subconjunctival hemorrhage with clear cornea. After five days, the mouth lesions began to heal with some residual erosions, white slough, crusting, and desquamation of the mucosal lips. He had perilimbal conjunctival healing but had a target lesion on the left lower lid palpebral conjunctiva. After seven days, the patient was able to tolerate oral intake. He had numerous dusky, violaceous macules, some with central bullae formation and others with crusted erosions. The patient was discharged after seven days. Two days post-discharge, the patient had a follow-up appointment with ophthalmology. He had ongoing erythema and skin peeling, moderate mattering, and conjunctival injection. The patient had some symblepharon in bilateral lids. The corneas were clear bilaterally with no significant staining. Approximately three and a half months post-discharge, he presented to the ophthalmologist with bilateral epiphora. Trichiasis was noted in the right lower eyelid. Bilaterally, he had symblepharon of the lower eyelids and punctal stenosis. Permanent complications include onychoschizia of the fingernails and toenails, skin hyperpigmentation of larger torso lesions, reduced range of motion of the tongue, bilateral trichiasis, and bilateral epiphora.

Initial laboratory work consisted of urine test and culture, complete blood count, complete metabolic panel, blood culture, and a viral direct detection test. The red blood cell count (4.39x10^-6^/mcL) and absolute lymphocyte count (0.60x10^-3^/mcL) were low. The erythrocyte sedimentation rate (60 mm/h) and c-reactive protein level (18.6 mg/dL) were high. Lab results showed, he had an elevated HSV-1 IgG (5.27 index) and *Mycoplasma pneumoniae* IgM (1.94 units/L) and IgG (2.24 units/L). No biopsies of the lesions were performed since a clinical diagnosis was made at the time.

While awaiting his laboratory results, the patient was initially treated with 10 mL oral viscous lidocaine, 10 mL of oral aluminum hydroxide-magnesium hydroxide suspension, and 20% benzocaine spray, in addition to 500 mg azithromycin and 700 mg acyclovir. These medications were continued when titer levels for *Mycoplasma pneumoniae *and HSV-1 came back elevated. Intravenous patient-controlled analgesics of 50 mcg/mL fentanyl were added to his therapy for pain control. Eyes were managed with artificial tears-ophthalmic ointment, lacri-lube, and erythromycin eye drops. Urinary discomfort, due to urethral meatus involvement, was managed with topical 2% lidocaine gel with an applicator. During the peak of the lesion outbreaks, 40 mg IV methylprednisolone was added to manage progression of cutaneous and mucosal lesions. Vaseline was applied to open lesions. The patient was also treated with mouthwash consisting of aluminum (Al) hydroxide/magnesium (Mg) hydroxide/simethicone (400 mg/400 mg/40 mg). Upon ophthalmology follow-up three and a half months post-discharge, he had bilateral punctoplasty.

On the day of his hospital discharge, the lesions were stable and the patient could tolerate oral intake. He was to continue topical therapy, oxycodone and prednisone taper. Post-hospital discharge, he continued to experience ocular sequelae including trichiasis, symblepharon, and punctal stenosis.

## Discussion

Erythema multiforme (EM) is a rare, acute inflammatory skin disease typically occurring in adults before 50 years of age [1]. EM is clinically divided into minor (absent/mild mucous membrane involvement) and major (one or more mucous membrane involvement) subtypes. Skin lesions are classically located on the extensor surfaces of acral extremities [1,7,9]. EM is now regarded as a distinct diagnosis from SJS/TEN. EM differs from SJS/TEN in etiology, clinical manifestations, histopathology, treatment, and prognosis [5,7,9].

Prodromal weakness, fever, and malaise commonly occur approximately one week before the onset of skin lesions [2]. In EM, there is a rapid onset of lesions, which may be relapsing in nature [1]. EM is characterized by the presence of target lesions with three concentric rings, some with epidermal changes, such as blistering or crusting, predominantly in an acral distribution. Atypical lesions seen in EM have been described as raised and edematous, with two zones of color change and a poorly defined border [1,7,9].

Ocular manifestations with EMM are rare, and range in severity. A case study conducted by Chang et al. studied 65 patients diagnosed with EMM. Out of the 65 patients, 15 had ocular manifestations. Mild involvement was the most common (86.7%) followed by moderate (6.7%) and severe (6.7%) involvement. In these cases, 93% presented with conjunctivitis, 13.3% with blepharitis, 6.7% conjunctival pseudomembrane, 6.7% conjunctival membrane, and 6.7% with symblepharon [10]. It should be noted that patients presented with either one or more of these manifestations. Pertinent ocular sequelae were documented three months after an initial attack occurred in 6.7% of cases and included dry eyes and symblepharon [10].

Up to 90% of cases of EM are associated with infectious causes, mainly HSV and *Mycoplasma pneumoniae* [9]. *Mycoplasma* infection is more commonly associated with EMM, while recurrent HSV infections are more associated with EMm [5]. There is an autoimmune component of EM, with etiologies tending to be unclear in many cases; however, a cytotoxic type-IV reaction, mediated by T lymphocytes, seems to be consistent throughout cases [1].

Diagnosing EMM can be challenging if there are atypical lesions present. Serology and biopsies can be useful in determining etiology. Both EMM and EMm have similar histological features, with the duration of the lesion affecting the extent of involvement of the epidermis. The level of epidermal involvement may also depend on the location of the biopsy within the lesion. Cellular necrosis is highly characteristic of all lesions produced by EMM and EMm. Biopsies of EMM show vacuolar interface reactions with a mononuclear cell infiltrate around vessels and the dermal-epidermal junction. In the lesion, there is a prominence of activated T lymphocytes [5,7]. There are cytotoxic and suppressors cells in the epidermis. Within the dermal layer, there are helper T cells. There is an absence of leukocytoclastic vasculitis and there are rarely any eosinophils present. The pathologic differential diagnosis includes fixed drug eruptions, graft versus host disease, pityriasis lichenoides, and lupus erythematosus [5].

Treatments of EMM should be based on the potential etiologies of patient symptoms [1]. If EM is drug-induced, it requires immediate removal of the offending agent and avoidance of similar chemical structured drugs [2,7]. Since most cases of EM are due to infectious etiologies, medications to target those agents are useful. Acyclovir is used for HSV-induced EM, while macrolides, like azithromycin, are typically used for *Mycoplasma pneumoniae*. Systemic corticosteroids are widely used to manage acute outbreaks [1]. Untreated outbreaks, if not severe, will pass on their own, but recovery occurs at a much slower rate than if corticosteroids, or other treatments targeting infectious causes, are administered [1]. The case described here had several potential etiologies. Therefore, the treatment with corticosteroids, acyclovir, and azithromycin proved beneficial. Topical prophylactic treatment with acyclovir does not seem to prevent recurrent HSV-associated EM [7].

This case highlights how several documented etiologies of EMM may occur simultaneously. When multiple factors are involved, the threshold for each to cause an EMM outbreak to occur may be lowered. Additionally, the triad of involved oral, ocular, and urethral mucosa is rarely documented in the literature. This triad of affected mucosal surfaces may be due to the combination of precipitating factors (NSAIDS, HSV-1, and *Mycoplasma pneumoniae*).

Classification of EM has been a highly debated topic as it is usually placed clinically within a spectrum of SJS/TEN; however, there is more evidence arising to distinguish it as its own distinct condition [8,9,11]. Diagnosis historically is based on clinical presentation, patient history, and the manifestations of cutaneous and/or mucosal lesions. A skin biopsy of the lesions can be helpful [1]. In future cases, all these things must be taken into consideration when diagnosing and treating EM/SJS/TEN.

Unique diagnosis and treatment challenges exist with patients diagnosed with EMM. By the time this patient presented to the ED, he had several lesions scattered over his body as well as eye involvement. Rapid collaboration of dermatologists and ophthalmologists was vital for the management of the patient's severe form of EMM. Treatment with antivirals, antibiotics, steroids, opioids, and eye lubrication was initiated prior to serology results, as these options covered all of the most common EMM etiologies. Once titers were obtained and confirmed HSV-1 and *Mycoplasma​​​​​​​ pneumoniae*, the treatment regimen was continued. This treatment resulted in the healing of the targetoid lesions, as well as the conjunctiva. Upon discharge, he continued to follow-up with dermatology and ophthalmology for continued care. Ultimately, this case supports multispeciality involvement. Due to this approach, the patient was able to regain all activities of daily living with minimal ocular complications.

## Conclusions

This case highlights the multifactorial etiologies of EM, including infection, drug-induced, and idiopathic forms. The severity of this case may be due to a combination of triggers, such as recent NSAID use, HSV-1 infection, and M*ycoplasma pneumoniae *infection. This emphasizes the importance of conducting a thorough history, physical examination, serology, and biopsies during atypical presentations of EM. These are all needed to determine the best treatment plan and multidisciplinary approach. Treatment for EM most commonly includes steroids for inflammation, pain management, and treatment of the underlying cause. Prompt recognition and treatment aided the reduction of long-term compilations. Future reports detailing treatment strategies, as well as affected body surfaces like ocular mucosal involvement are also useful for clinicians. This will assist in monitoring and preventing symptom progression so long-term sequelae can be avoided and hospital stays can be reduced.
